# SNARE Proteins LjVAMP72a and LjVAMP72b Are Required for Root Symbiosis and Root Hair Formation in *Lotus japonicus*

**DOI:** 10.3389/fpls.2018.01992

**Published:** 2019-01-16

**Authors:** Aoi Sogawa, Akihiro Yamazaki, Hiroki Yamasaki, Misa Komi, Tomomi Manabe, Shigeyuki Tajima, Makoto Hayashi, Mika Nomura

**Affiliations:** ^1^Faculty of Agriculture, Kagawa University, Kagawa, Japan; ^2^RIKEN Center for Sustainable Resource Science, Yokohama, Japan

**Keywords:** *Lotus japonicus*, nitrogen fixation, root hair, SNARE, symbiosis, VAMP72

## Abstract

SNARE (soluble N-ethyl maleimide sensitive factor attachment protein receptor) proteins mediate membrane trafficking in eukaryotic cells. Both LjVAMP72a and LjVAMP72b are members of R-SNARE and belong to a symbiotic subgroup of VAMP72 in *Lotus japonicus*. Their sequences are closely related and both were induced in the root upon rhizobial inoculation. The expression level of *LjVAMP72a* in the nodules was higher than in the leaves or roots; however, *LjVMAP72b* was expressed constitutively in the leaves, roots, and nodules. Immunoblot analysis showed that not only LjVAMP72a but also LjVAMP72b were accumulated in a symbiosome-enriched fraction, suggesting its localization in the symbiosome membrane during nodulation. Since there was 89% similarity between LjVAMP72a and LjVAMP72b, knockdown mutant by RNAi suppressed both genes. The suppression of both genes impaired root nodule symbiosis (RNS). The number of bacteroids and the nitrogen fixation activity were severely curtailed in the nodules formed on knockdown roots (*RNAi-LjVAMP72a/72b*). Arbuscular mycorrhization (AM) was also attenuated in knockdown roots, indicating that *LjVAMP72a* and *LjVAMP72b* were required to establish not only RNS but also AM. In addition, transgenic hairy roots of *RNAi-LjVAMP72a/72b* suppressed the elongation of root hairs without infections by rhizobia or arbuscular mycorrhizal fungi. Amino acid alignment showed the symbiotic subclade of VAMP72s containing *LjVAMP72a* and *LjVAMP72b* were a conserved six amino acid region (HHQAQD) within the SNARE motif. Taken together, our data suggested that LjVAMP72a and LjVAMP72b positively controlled both symbioses and root hair formation by affecting the secretory pathway.

## Introduction

Legume-rhizobia interaction has developed from complex signal exchange. Upon sensing host plant-derived flavonoids, rhizobia produce and secrete lipochito-oligosaccharides called nodulation factors (nod factors), which are then recognized by the host and trigger multiple events leading to establishing root nodule symbiosis (RNS) (Dénarié et al., 1996; Oldroyd, 2013). During nodulation, bacterial infection and nodule organogenesis take place in epidermal and cortical cells, respectively (Suzaki and Kawaguchi, 2014). Nod factors trigger deformation and curling of root hairs. Rhizobia then penetrate into root hairs via infection threads (Kouchi et al., 2010; Oldroyd, 2013). Simultaneously, cell division occurs in the cortical layer to form nodule primordia (Oldroyd, 2013). When infection threads reach the nodule primordia, rhizobia are released from the infection threads to nodule primordia by an endocytosis-like process, in which rhizobial cells are surrounded by host-derived membranes [symbiosome membrane (SM)] called a “symbiosome” (Newcomb, 1976; Verma et al., 1978). In addition to RNS, legumes establish an endosymbiotic association with arbuscular mycorrhizal fungi (AMF). AMF secrete sulfated and non-sulfated lipochito-oligosaccharide signals (Myc-LCOs), which function as signals for allowing AMF to enter host roots (Maillet et al., 2011). AMF penetrate the root epidermis by forming hyphopodia on the root surface and extend their internal hyphae toward the inner cortex, where they form arbuscules that are surrounded by a plant plasma membrane called a periarbuscular membrane (PAM). These dynamic changes to form a SM and PAM are closely associated by membrane trafficking of endoplasmic reticulum (ER) and/or Golgi apparatus and their transport vesicles (Robertson et al., 1978; Newcomb and McIntyre, 1981; Roth and Stacy, 1989; Genre et al., 2012).

SNARE (soluble NSF attachment protein receptor) proteins are known to mediate the transport vesicle, and its molecules have a highly conserved coiled-coil domain known as a SNARE motif (Harbury, 1998; Jahn et al., 2003). The SNARE-mediated membrane trafficking forms SNARE complexes with R-SNARE on the vesicle and two or three Q-SNAREs on the target membrane. During the past decade, it has been clear that SNARE proteins play roles in general homeostatic and housekeeping functions within the cell. In *Arabidopsis*, two plasma membrane-localized Qa-SNAREs, SYP123 and SYP132, mediate membrane trafficking for root hair formation (Ichikawa et al., 2014). Recently, a symbiotic Qa-SNARE protein, SYP132A, has been shown to be generated by alternative termination of transcription and is essential for the formation of both SM and PAM in *Medicago truncatula* (Huisman et al., 2016; Pan et al., 2016). R-SNAREs located on the transport vesicles are classified into three groups: Sec22, YKT6, and VAMP7 (McNew et al., 1997; Galli et al., 1998; Gonzalez et al., 2001). VAMP7 consists of two major subgroups in plants: VAMP71 and VAMP72 (Sanderfoot, 2007). VAMP71 is known to have a similar function to mammalian VAMP7s; however, another group, VAMP72, appears to be specific to green plants (Sanderfoot, 2007). The *Arabidopsis* VAMP72 proteins except AtVAMP727 are localized in the plasma membrane (Marmagne et al., 2004; Uemura et al., 2004). To determine the specific function for symbiosis, it is better to focus on *VAMP72* in legume plants. In *M. truncatula*, VAMP721d and VAMP721e are required for both RNS and arbuscular mycorrhization (AM) (Ivanov et al., 2012). For *Lotus japonicus*, there are reports on SNARE-mediated membrane trafficking in nodules. The sed5-like *LjSYP132-1*, Qa-SNARE, contributes to nodule formation and plant growth development (Mai et al., 2006). *SYP71*, Qc-SNARE, plays a role in symbiotic nitrogen fixation (Hakoyama et al., 2012). However, there is no report on R-SNARE. In this study, we focused on an R-SNARE, *VAMP72*, in the formation of symbiotic interfaces. Our data revealed that *LjVAMP72a* and *LjVAMP72b* were required for both RNS and AM as well as for root hair development.

## Materials and Methods

### Plant and Microbial Materials

Seeds of *L. japonicus* B129 Gifu (Handberg and Stougaard, 1992) were surface sterilized with 3% sodium hypochlorite and then germinated. Six-day-old seedlings were transplanted onto sterile vermiculite with a half concentration of B&D medium (Broughton and Dilworth, 1971), followed by inoculation with *Mesorhizobium loti* strain MAFF303099 at 7 days after transplanting. Plants were grown in a growth chamber at 24°C under a 16 h light/b h dark condition.

### RNA Extraction and Real-Time PCR Analysis

Total RNA was isolated from various organs of *L. japonicus* using the RNeasy Plant Mini Kit according to the manufacturer’s instructions (Qiagen, Tokyo, Japan). The cDNA was synthesized using PrimeScript RT Master Mix (Takara, Shiga, Japan). Quantitative RT-PCR reactions were performed in triplicate on cDNA (equivalent to about 100 ng of RNA) using the SYPR premix Ex Taq (Takara), and real time detection was performed on a Thermal Cycler Dice Real Time System II (Takara) with the primer set shown in Supplementary Table S1. Ubiquitin was used as an internal standard. All data were normalized because the expression levels of ubiquitin are equal in each sample. All experiments were performed with more than three biological replications.

### Orthology Prediction and Phylogeny Reconstruction

Putative orthologs of *MtVAMP72d* were searched for in a database consisting of genomes and transcriptomes (i.e., all available CDSs) of 70 green plant species (Sato et al., 2008; Carleton College, and The Science Education Resource Center, 2010; Goodstein et al., 2012; Dash et al., 2016) (see Supplementary Table S1 for the complete list). OrthoReD (Battenberg et al., 2017) was used to predict the orthologs of MtVAMP72d with its amino acid sequence as a query against the database without setting any outgroups. Based on the phylogenetic tree reconstructed by OrthoReD, a clade of putative orthologs of MtVAMP72d in several species (*A. thaliana*, *Glycine max*, *L. japonicus*, *M. truncatula*, *Solanum lycopersicum*, *Populus trichocarpa*, and *Vitis vinifera* following Ivanov et al., 2012) was determined manually. Gene names corresponding to Gene ID used in this study were listed in Supplementary Table S2.

The phylogeny of these putative orthologs was reconstructed. Amino acid sequences of these putative orthologs were aligned using MAFFT v7.407 (Katoh and Standley, 2013). Based on the multiple sequence alignment, the most likely phylogeny was reconstructed using RAxML v8.2.12 (Stamatakis, 2006). The best of four parallel runs was chosen as the most likely tree to avoid having the tree lodged onto a local optimum. Rapid bootstrapping of 100 replicates was also carried out using RAxML v8.2.12.

### DNA Sequencing

Isolated cDNA and PCR amplified products were sequenced according to the manufacturer’s instructions for the DNA sequencer (Model 3700; Applied Biosystems, CA, United States). The CDS of *LjVAMP72b* was speculated by genome sequence (Sato et al., 2008) and nodule cDNA was amplified with following primers; FW, 5′-ATGGGTCAGAACCAGAGATC-3′; RV, 5′-CCTTTCCTCGCATAATCACAA-3′ using Tks Gflex DNA polymerase (Takara, Japan). The resulting PCR product was sequenced from both strands.

### Construction of *LjVAMP72a prom::GUS*, *RNAi-LjVAMP72a/72b*, and *LjVAMP72a-GFP*

To construct the *LjVAMP72a* promoter fused to the *GUS* gene, the *GUS-NOS* gene was first cloned into the *Xba*I/*Sal*I sites of p*C1300GFP*, kindly provided by Dr. H. Kouchi (National Institute of Agricultural Sciences, Tsukuba), producing the *GUS-NOS in pC1300 GFP*. The 5′-flanking regions [-1,817 to before ATG of the translation-initiation site in the *LjVAMP72a* gene (genome clone; CM0066)] was then cloned into the *GUS-NOS in pC1300GFP*, following the production of *LjVAMP72a prom::GUS*.

To construct *RNAi-LjVAMP72a/72b*, the cDNA of *LjVAMP72a* was amplified using PCR from an EST clone MR100a03 (accession No. BP083622) with the following primers: RNAi-f, 5′-ATG GAT CCC TCG AGA ATG GCT ACA CAT ATT-3′ and RNAi-r, 5′-ATA TCG ATG GTA CCT TTT GGG AAT CCA CGA-3′. The amplified cDNA fragments for the RNAi construct was subcloned into the appropriate cloning sites of *pC1300GFP*. The procedure for constructing it has been described previously (Shimomura et al., 2006). Since the amplified cDNA has high similarity between *LjVAMP72a* and *LjVAMP72b*, the knockdown mutant suppressed both genes. Therefore the mutant was named as *RNAi-LjVAMP72a/72b*.

To construct *LjVAMP72a-GFP*, used by *Arabidopsis* suspension cells, *LjVAMP72a* cDNA was fused to the *sGFP* under the control of CaMV35S-sGFP (S65T)-NOS3′ (Niwa et al., 1999), provided by Dr. Y. Niwa, University of Shizuoka.

### Hairy Root Transformation

Hairy root transformation of *L. japonicus* Gifu using *Agrobacterium rhizogenes* LBA1334 was performed according to a procedure described previously (Kumagai and Kouchi, 2003). Transgenic hairy roots with GFP fluorescence were selected and transferred to vermiculite pots with a half concentration of B&D medium. Plants were grown in a growth cabinet at 24°C (16 h light/b h dark). After 1 week, the plants were transferred to pots and then inoculated with *M. loti* MAFF303099 and continued to grow in the same medium.

### Histochemical GUS Staining

Hairy roots transformed with the *LjVAMP72a prom::GUS* construct were stained for 2 h at 37°C as described previously (Mai et al., 2006). GUS-stained nodules were observed using a DM6 upright microscope (Leica, Germany) with a differential interference contrast mode. Mycorrhized roots were embedded in 5% agarose, sliced in b0 μm sections using a Zero 1 super microslicer (D.S.K., Japan), and observed using the DM6 (Leica).

### Transient Expression of the GFP-Fused Protein, LjVAMP72a-GFP, in *Arabidopsis* Suspension Cells

*LjVAMP72a* cDNA was fused to the *sGFP* of a CaMV35S-sGFP (S65T)-NOS3′ vector, producing the *LjVAMP72a-GFP*. RFP-tagged *Arabidopsis AtSYP722*, *AtSYP722-RFP*, was kindly provided by Dr. T. Ueda, University of Tokyo. The transient expression of LjVAMP72a-GFP and AtSYP722-RFP in *Arabidopsis* suspension culture cells was performed following a previous report (Ueda et al., 2001).

### Detection of LjVAMP72a and LjVAMP72b Proteins

Plant tissue (0.1 g) was harvested after 35 days infection and homogenized with a mortar and pestle in extraction buffer containing 250 mM Tris-HCl (pH 7.5), 700 mM sucrose, 100 mM KCl, 2% (w/v) insoluble polyvinylpolypyrrolidone, 2% 2-mercaptoethanol, and 2% SDS. After total maceration, water-saturated phenol was added and then centrifuged at 3,000 ×*g* for 20 min. The phenol fraction was isolated and five volumes of methanol plus 100 mM ammonium acetate including 10 mM 2-mercaptoethanol were added. After incubation at -80°C for 2 h, the solution was centrifuged at 15,000 ×*g* for 5 min, and the pellets were resuspended with sample buffer [50 mM Tris-HCl (pH 6b), 100 mM DTT, 2% SDS, and 10% glycerol]. SDS-polyacrylamide gel electrophoresis (SDS-PAGE) was performed according to the procedure of Nomura et al. (2006). Immunoblot analysis was performed with rabbit antisera raised against a protein of (ENIEKVLDRGEKIE) antibody as the primary antibody and detected using ECL and a western blotting detection system (GE Healthcare, United Kingdom). Since the produced antibody can detect both proteins, the resulting antibody was named anti-LjVAMP72a/72b antibody. The protein concentration was determined using the Lowry method (Lowry et al., 1951). Lb antibody raised against rabbit was kindly provided by Prof. N. Suganuma, Aichi University of Education, Japan.

### Isolation of Symbiosome Fraction From the Nodules

Fresh nodules (10–15 g) at 4 weeks post infection were used to isolate the symbiosome fraction. The symbiosome fraction was isolated from Percoll discontinuous gradients (Day et al., 1989). In brief, nodule homogenate was centrifuged at 1,000 rpm for 15 min, and the pellet was then suspended with washing medium and further centrifuged at 2,000 rpm for 10 min. The resulting pellet was used for Percoll discontinuous gradients (80, 60, and 45%), and loose pellets were collected from the bottom of the 80% layer and the 60/80% interface as a symbiosome fraction. The isolated symbiosome fraction was suspended in washing medium and used for immunoblot analysis. After centrifugation at 1,000 and 2,000 rpm, both supernatants were used as a soluble fraction.

### Assay for Acetylene Reduction

For acetylene reduction activity, transgenic hairy root nodules were selected under fluorescence microscopy because the GFP gene was inserted in the construct vectors. The activity of GFP-positive nodules was measured using gas chromatography (Shimadzu GC-8A; Kyoto, Japan) as previously described (Suganuma et al., 1998). Experiments were performed with more than three biological replications.

### Root Hair Phenotype in *RNAi-LjVAMP72a/72b* Knockdown Roots

Hairy roots of *L. japonicus* expressing the *LjVAMP72a* and *LjVAMP72b* knockdown construct (*RNAi-LjVAMP72a/72b*) were observed using the DM6 (Leica). Pictures were tiled using Microsoft Image Composite Editor (Microsoft, United States). The length of the root hairs was measured from a position 1 mm from the root tip. The length of 50 root hairs per transgenic plant was measured using Image J. Experiments were performed with more than three biological replications.

### Mycorrhization Assay

*Lotus japonicus* expressing the *RNAi-LjVAMP72a/72b* construct or *GFP* only (empty vector) in the hairy roots were planted in plastic pots filled with sterile vermiculites in ½ Hoagland culture medium and inoculated with *Rhizophagus irregularis* (Premier Tech, Rivière-du-Loup, Canada) at 100 spores/plant. At 6 weeks after inoculation, the roots were stained with 0.05% trypan blue, and AM events were numbered and calculated using the line intersect method (McGonigle et al., 1990). Pictures of AMF-inoculated roots were taken using the DM6 (Leica) after staining.

### Analysis of Nodulation and Bacteroid Number in Transgenic Hairy Roots

Hairy roots were inoculated with *M. loti* strain MAFF303099 expressing a red fluorescent protein from *Discosoma* sp. (DsRed) (Maekawa et al., 2009) and used in our nodulation test. Transgenic hairy roots were selected by GFP fluorescence and observed under a fluorescence microscope (Olympus SZX9). After infection of DsRed-labeled *M. loti*, bacteroid was isolated from the nodule and the number of bacteroid in each transgenic nodule was counted using fluorescent microscope BX51 (Olympus).

## Results

### *LjVAMP72a* and *LjVAMP72b* Were Induced Upon Rhizobial Inoculation

**FIGURE 1:**
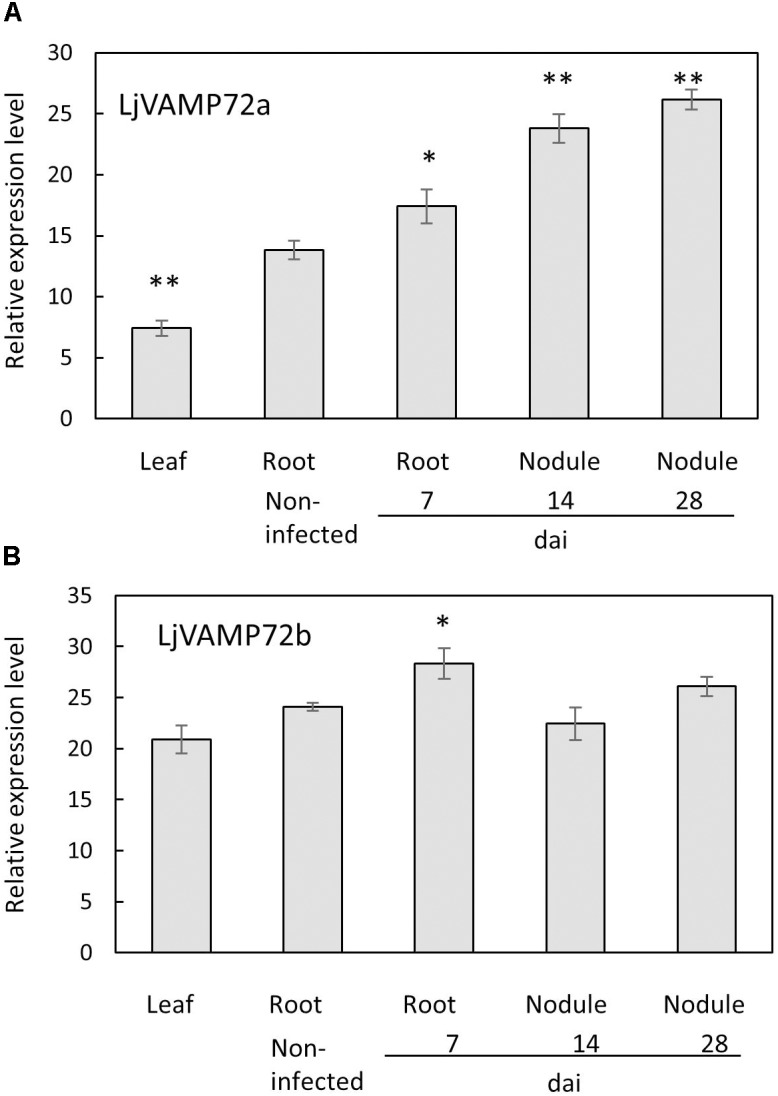
**(A,B)** Expression of *LjVAMP72a* and *LjVAMP72b* mRNA after inoculation with *M. loti*. The ubiquitin was used as an internal control. dai: days after inoculation. Experiments were performed with more than three biological replications. Statistically significant differences compared with non-infected roots were indicated by asterisks (^∗^*p* < 0.05, ^∗∗^*p* < 0.001).

The genome database of *L. japonicus*^1^ possesses several VAMP72 proteins from LjVAMP72a to LjVAMP72f, LjVAMP724 and LjVAMP727. We found that one of the LjVAMP72s, *LjVAMP72a* in *L. japonicus*, was expressed in the nodules, induced upon rhizobial inoculation, and peaked at 14 days after inoculation (Figure 1). In addition, the genome database contains another *LjVAMP72*, *LjVAMP72b*, which has high homology to *LjVAMP72a*. There was 89% similarity between LjVAMP72a and LjVAMP72b. The expression of the *LjVAMP72a* genes was induced in the infected roots and nodules; however, *LjVAMP72b* was expressed constitutively in the leaves, roots, and nodules (Figure 1).

**FIGURE 2:**
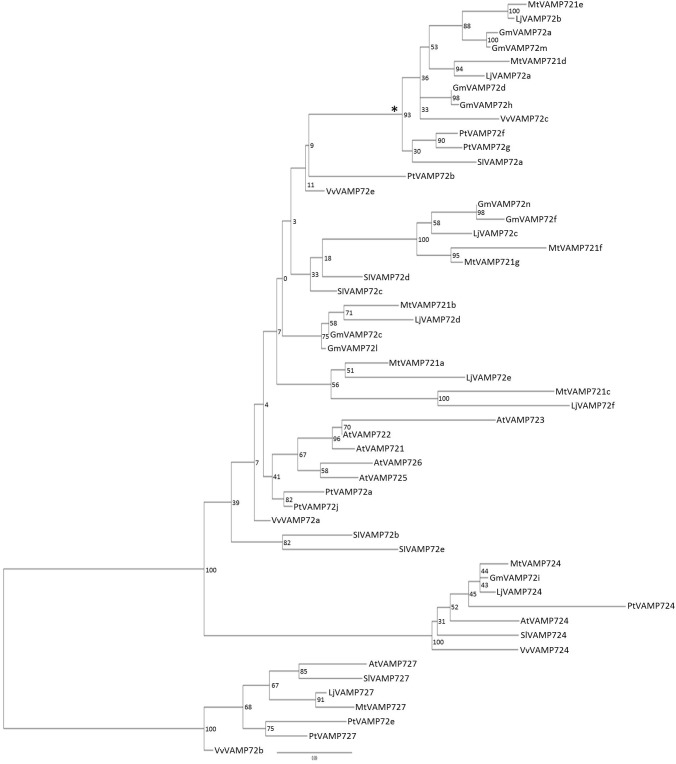
Phylogenetic tree of VAMP72s. Phylogenetic tree of VAMP72s among *L. japonicus* (LjVAMP72s), *Medicago truncatula* (MtVAMP72s), *G. max* (GmVAMP72s), *Phaseolus vulgaris* (PvVAMP72s), *Vitis vinifera* (VvVAMP72s), *Populus trichocarpa* (PtVAMP72s), *Solanum lycopersicum* (SlVAMP72s), and *Arabidopsis thaliana* (AtVAMP72s). Gene ID corresponding to gene name was listed in Supplementary Table S2. The symbiotic subgroup was indicated with an asterisk. AtVAMP727 was used as an outgroup.

The phylogenic analysis (Figure 2) showed that both LjVAMP72a and LjVAMP72b belong to the “symbiotic” subgroup and were orthologous to MtVAMP721d and MtVAMP721e, respectively, which have been reported to function in symbioses (Ivanov et al., 2012).

**FIGURE 3:**
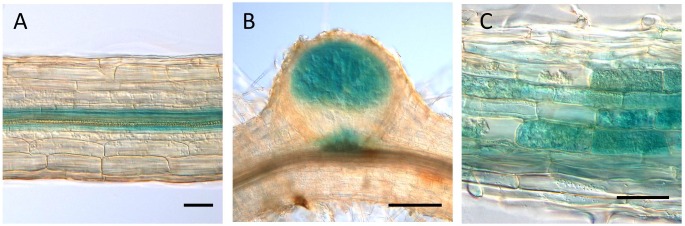
*LjVAMP72a* promoter activity during symbioses. The promoter activity of LjVAMP72a was visualized by staining the β-glucuronidase activity in transgenic hairy roots under three different conditions: **(A)** non-inoculated, **(B)** inoculated with *M. loti* MAFF303099, and **(C)** inoculated with *R. irregularis*. Pictures were taken at 14 and 48 days after inoculation for **(B,C)**, respectively. Scale bars represent 100 μm for **(A,C)** and 200 μm for **(B)**.

The GUS expression by the *LjVAMP72a* promoter was detected at the vascular bundle in non-infected roots (Figure 3A). The expression was strongly induced in nodule primordia upon rhizobial inoculation besides its basal expression at the vascular bundles of the roots (Figure 3B). The GUS induction at the vascular bundle of infected roots was detected with longer staining (data not shown). The higher GUS expression by the *LjVAMP72a* promoter in the nodules than that in the roots was identical to that expression level of the *LjVAMP72a* in nodules was higher than that in roots at 28 days after infection (Figure 1). AM also positively affected *LjVAMP72a* promoter activity. During AM, *LjVAMP72a* was induced in cells where arbuscules formed (Figure 3C). These data suggested that the *LjVAMP72a* plays a role in both symbioses, RNS and AM. Because the genome sequence of *LjVAMP72b* was not completed, we have not constructed the *LjVAMP72b* promoter fused to the GUS gene.

### Localization of LjVAMP72a and LjVAMP72b in RNS

**FIGURE 4:**
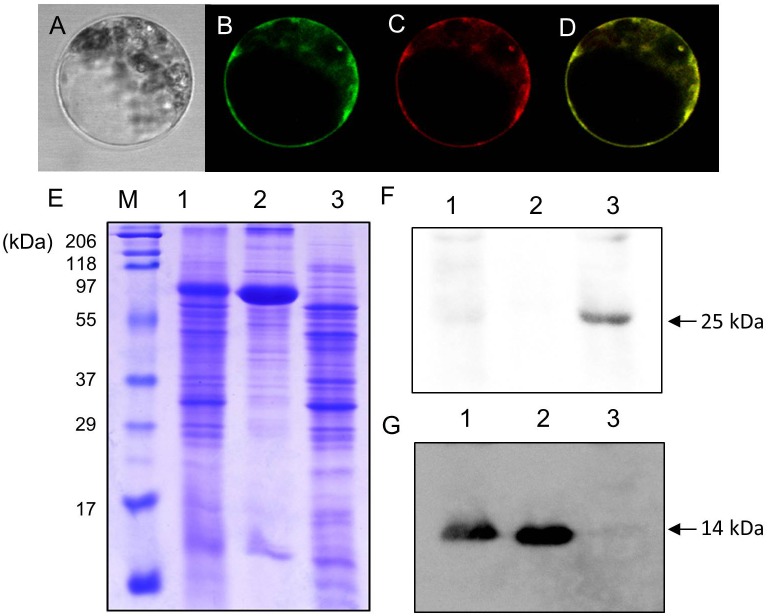
Cellular localization of LjVAMP72a and LjVAMP72b. **(A–D)** Transient expression of LjVAMP72a in *Arabidopsis* suspension cells. The GFP-tagged LjVAMP72a **(B)** and RFP-tagged *Arabidopsis* AtSYP722 **(C)** were transiently expressed in the protoplasts of *Arabidopsis* cells. Bright field **(A)** and fluorescence images were captured from the same cells expressing each GFP-tagged LjVAMP72a **(B)** or RFP-tagged AtSYP722 **(C)**. **(D)** is a merging of **(B,C)**. **(E)** Fractionated extracts from nodules were separated by SDS-PAGE and stained with CBB. **(F,G)** Immunoblot analysis using anti-LjVAMP72a/72b antibody **(F)** or anti-Lb antibody **(G)**. Lane 1: crude extract from nodules, lane 2: supernatant of the crude extract, lane 3: symbiosome-enriched fraction of a Percoll-mediated equilibrium density-gradient centrifugation. Arrowheads indicate LjVAMP72a/72b **(F)** and Lb **(G)**, respectively.

Uemura et al. (2004) identified AtVAMP722 SNARE localized in the plasma membrane in *Arabidopsis* suspension cells. Figure 4 shows LjVAMP72a co-localization with AtVAMP722 in suspension culture cells of *A. thaliana*, indicating its localization in plasma membrane (Figures 4A–D). We have produced a peptide antibody that can detect both LjVAMP72a and LjVAMP72b. After isolating the symbiosome-enriched fraction, immunoblot analysis was performed (Figure 4). Since the deduced molecular weight of LjVAMP72a and LjVAMP72b was similar (ca. 25 kDa), one band was detected at the deduced molecular size in the symbiosome-enriched fraction (Figure 4F, Lane 3). The LjVAMP72a and LjVAMP72b were not fractionated in the soluble fractions in which leghaemoglobin was detected (Figure 4G, Lanes 1 and 2). SM contains some of components present in the plasma membrane and the results showing its localization in the plasma membrane were obtained in *Arabidopsis* suspension cells. Taken together, these data indicated that LjVAMP72a and LjVAMP72b localized in the SM.

### LjVAMP72a and LjVAMP72b Functioned in Symbioses

**FIGURE 5:**
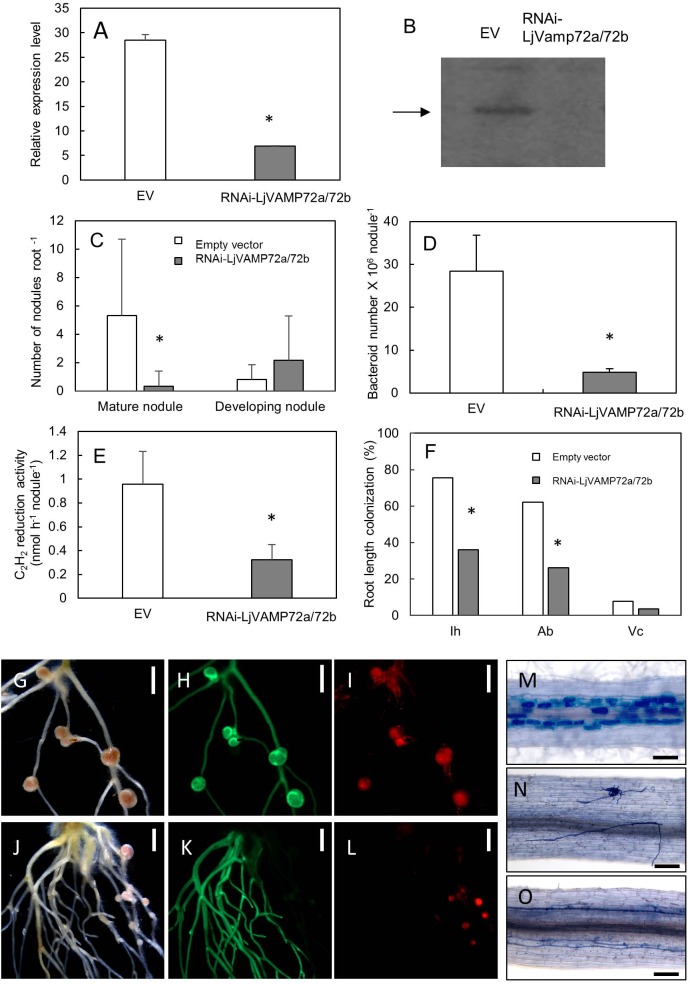
LjVAMP72a and LjVAMP72b were required for RNS and AM. RNA interference of *LjVAMP72a/72b* was confirmed using qRT-PCR **(A)** and western blot using an anti-LjVAMP72a/72b antibody **(B)**. Arrowhead indicates LjVAMP72a. LjVAMP72a/72b knockdown attenuated the number of mature nodules **(C)**, the number of bacteroids **(D)**, acetylene reduction activity **(E)**, and arbuscular mycorrhization **(F)**. Photos of nodulation and arbuscular mycorrhization **(G–O)**. Pictures of *M. loti* DsRed13-inoculated hairy roots transformed with an empty vector **(G–I)** or RNAi-LjVAMP72a/72b **(J–L)** at 28 days after inoculation were taken with a bright field **(G,J)**, GFP fluorescence **(H,K)**, and DsRed fluorescence **(I,L)**. Transgenic hairy roots transformed with an empty vector **(M)** or with RNAi-LjVAMP72a/72b **(N,O)** were inoculated with *R. irregularis* and were stained with toluidine blue at 42 days after inoculation. The square boxes of white and gray in **(C,F)** indicate transgenic hairy roots transformed empty vector and RNAi-LjVAMP72a/72b, respectively. EV, empty vector; Ih, internal hyphae; Ab, arbuscule; Vc, vesicle. Scale bars represent 2 mm for **(G–L)** and 100 μm for **(M–O)**. All experiments were performed with more than three biological replications. Statistically significant differences compared with EV are indicated by asterisks (*p* < 0.01).

We made the transgenic hairy roots of knockdown *LjVAMP72a* genes. RNA interference of LjVAMP72a was confirmed using qRT-PCR (Figure 5A). Immunoblot analysis showed that no band could be detected in a knockdown mutant, suggesting that both LjVAMP72a and LjVAMP72b were suppressed in the knockdown mutant (Figure 5B). Therefore, the knockdown mutant was named as *RNAi-LjVAMP72a/72b*.

The number of mature nodules was significantly lower in the *RNAi-LjVAMP72a/72b* roots compared with that in the roots transformed with an empty vector (Figures 5C,G–L). On the other hand, the number of developing nodules of *RNAi-LjVAMP72a/72b* increased, and the number of bacteroids and acetylene reduction activity were severely attenuated in the *RNAi-LjVAMP72a/72b* roots (Figures 5D,E).

*LjVAMP72a* and *LjVAMP72b* also positively affected AM (Figures 5F,M–O). Frequencies of the internal hyphae invasion, arbuscular and vesicle formation were significantly impaired in the *RNAi-LjVAMP72a/72b* knockdown roots (Figure 5F). Figure 5N shows the external hyphae did not invade the roots. Some of the infected roots only exhibited internal hyphae with almost no arbuscule when *LjVAMP72a/72b* was knocked-down (Figure 5O), whereas the empty vector control barely affected arbuscule formation (Figure 5M). These data speculate the symbiotic membranes would be developed by the vesicle trafficking through the LjVAMP72a and LjVAMP72b, resulting that the *RNAi-LjVAMP72a/72b* knockdown attenuated symbioses. These results were in line with a previous report in which the VAMP721d and VAMP721e double knockdown attenuated the number of nodules in *M. truncatula* (Ivanov et al., 2012).

### *LjVAMP72a* and *LjVAMP72b* Positively Controls the Elongation of Root Hairs

**FIGURE 6:**
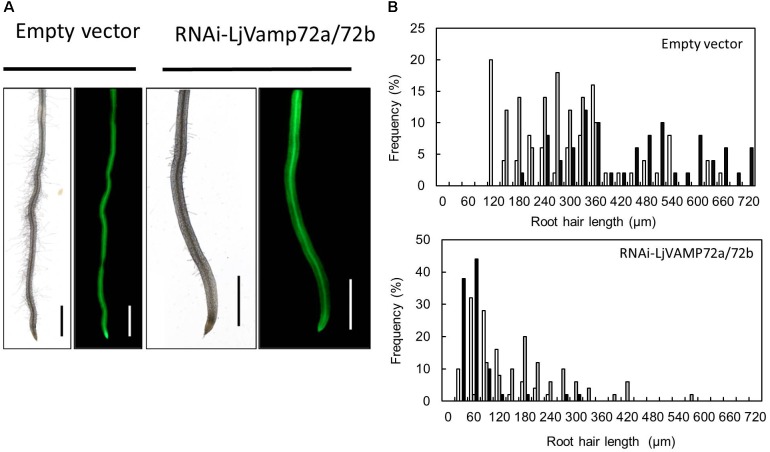
*LjVAMP72a* and *LjVAMP72b* functioned in the development of root hairs. **(A)** Transgenic hairy roots expressing either an empty vector or RNAi-LjVAMP72a/72b were subjected to microscopic observation for bright field (left) and GFP fluorescence (right). Scale bars represent 1 mm. **(B)** Histogram of root hair length in each transgenic hairy root. Upper and lower panels show transgenic lines transformed with empty vector and RNAi-LjVAMP72a/72b, respectively. Different colors indicate independent transgenic hairy roots.

Suppression of *LjVAMP72a* and *LjVAMP72b* mRNAs also affected the elongation of root hairs. *RNAi-LjVAMP72a/72b* knockdown roots exhibited significantly fewer root hairs or almost root hair-less phenotypes (Figure 6), suggesting that LjVAMP72a and LjVAMP72b functioned not only in symbioses but also in the development of root hairs.

### Amino Acid Sequence Alignment Conserved for the Symbiosis

**FIGURE 7:**
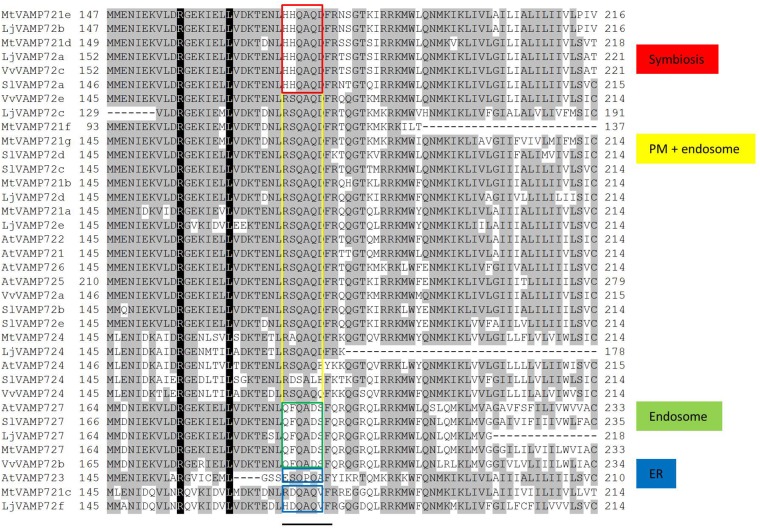
Sequence alignment of the VAMP72 group in plants. Conserved residues are shaded in black. A bar under the alignment represents the regions of possible localization signals (Uemura et al., 2004). Red boxes are conserved in the symbiotic VAMP72 subgroup.

SNARE proteins have a highly conserved coined-coil domain, called a SNARE motif (Harbury, 1998). Uemura et al. (2004) found a six amino acid region that could be classified by its subcellular localization within the SNARE motif. Figure 7 shows the sequence alignment of the VAMP72 groups. The aligned sequences were selected from legume plants, non-legume plants including *A. thaliana*. The alignment shows that the six amino acid regions (RSQAQD and QFQADS) of VAMP72s in *L. japonicus* were identical to the AtVAMP72s that localize in plasma membrane and endosome, respectively. These common six amino acid regions were detected in other species: *M. truncatula*, *A. thaliana*, *S. lycopersicum*, and *Vitis vulgaris*. A different six amino acid region (HHQAQD) was conserved in the “symbiotic” VAMP72 subgroup, including LjVAMP72a and LjVAMP72b (Figure 7).

## Discussion

For membrane trafficking, VAMP72 of R-SNARE is specific to green plants (Sanderfoot, 2007). Based on genome sequence information (Sato et al., 2008), rhizobia-induced *LjVAMP72a* and *LjVAMP72b* are classified in the symbiotic VAMP72 subgroup.

The producing knockdown mutant (*RNAi-LjVAMP72a/72b*) was suppressed both expressions (Figure 5B). In addition, RNS and AM were severely reduced on the *LjVAMP72a/72b* knockdown roots (Figure 5) and LjVAMP72a and LjVAMP72b were enriched in the symbiosome-containing fraction (Figure 4F), so LjVAMP72a and LjVAMP72b might be involved in the formation of a SM as reported in *M. truncatula* (Ivanov et al., 2012).

Both *LjVAMP72a* and *LjVAMP72b* were expressed in the nodules. The only difference was that the expression level of *LjVAMP72a* in the nodules was higher than that in the leaves or roots; however, *LjVAMP72b* was expressed constitutively in the leaves, roots, and nodules (Figure 1). A slight induction of *LjVAMP72b* appeared in the infected roots at 7 days compared with the non-infected roots. The data suggest that the expression ratio of *LjVAMP72a* and *LjVAMP72b* plays a role in forming the infection thread and the following SM. More data is needed to determine the different functions of the two.

Uemura et al. (2004) speculated that the six amino acid region within the SNARE motif differs depending on the subcellular localization in *Arabidopsis* VAMP72 proteins. From the genome data base, there were eight VAMP 72 genes in *L. japonicus* (Figure 2). In the alignment analysis (Figure 7), we could speculate the membrane localization of these Vamp72s from six amino acid region within the SNARE motif. Moreover, we found the symbiotic subgroup containing LjVAMP72a and LjVAMP72b conserved a different six amino acid region (HHQAQD). The symbiotic subgroup includes to non-legume genes, such as SlVAMP72a and VvVAMP72c (Figure 2, Ivanov et al., 2012) in *S. lycopersicum* and *V. vulgaris*, respectively, indicating that the *VAMP72s* with the six amino acid region (HHQAQD) is conserved among the R-SNARE for the localization of AM. The development of SM by LjVAMP72a and LjVAMP72b would be recruited during the evolution. The localization of another VAMP72s in *L. japonicus* (Figure 7) could be speculated from the six amino acid region that of AtVAMP72s localizing in the plasma membrane or endosome, except LjVAMP72f. The LjVAMP72f has a higher similarity to MtVAMP72c; however, there was no conserved amino acid in AtVAM72s of *A. thaliana*. Future studies may give more insight into the localization of LjVAMP72f.

We also found that LjVAMP72a/72b knockdown roots exhibited impaired root hair elongation (Figure 6), which was not reported in *M. truncatula* (Ivanov et al., 2012). Root hairs normally emerge at the apical end of root epidermal cells, implying that these cells are polarized because of vesicle targeting (Enami et al., 2009). In *Arabidopsis*, VAMP721/722/724 localizes in root hairs, and these AtVAMP72s form SNARE complexes with Q-SNARE, SYP123, and SYP132 (Ichikawa et al., 2014). LjVAMP72a and LjVAMP72b belonging to VAMP721 would have dual functions of root hair development and symbiotic membrane formation. Therefore, the Q-SNAREs responsible for root hair development and symbiotic membrane formation need to be confirmed. Taken together, our data suggested that the LjVAMP72a and LjVAMP72b played a role in RNS and AM as well as root hair development. Dual physiological activity of LjVAMP72a and LjVAMP72b likely involves a change in the vesicle cargo during the transition from plant growth to the symbiosis stage.

## Author Contributions

AS and AY performed most of the experiments and contributed equally. MH and MN designed the experiments. HY, MK, TM, and ST participated in some part of the study.

## Conflict of Interest Statement

The authors declare that the research was conducted in the absence of any commercial or financial relationships that could be construed as a potential conflict of interest.

## Footnotes

1 http://www.kazusa.or.jp/lotus/
